# Endemic erythromycin resistant *Corynebacterium diphtheriae* in Vietnam in the 1990s

**DOI:** 10.1099/mgen.0.000861

**Published:** 2022-10-19

**Authors:** To Nguyen Thi Nguyen, Christopher M. Parry, James I. Campbell, Phat Voong Vinh, Rachel Kneen, Stephen Baker

**Affiliations:** ^1^​ The Hospital for Tropical Diseases, Wellcome Trust Major Overseas Programme, Oxford University Clinical Research Unit, Ho Chi Minh City, Vietnam; ^2^​ Department of Microbiology, Monash University, Melbourne, Victoria, Australia; ^3^​ Centre for Tropical Medicine and Global Health, Nuffield Department of Medicine, University of Oxford, Oxford, UK; ^4^​ Clinical Sciences, Liverpool School of Tropical Medicine, Pembroke Pl, Liverpool L3 5QA, UK; ^5^​ Alder Hey Children’s Hospital, NHS Foundation Trust, Liverpool, UK; ^6^​ Institute of Infection and Global Health, University of Liverpool, Liverpool L69 3BX, UK; ^7^​ University of Cambridge School of Clinical Medicine, Cambridge Biomedical Campus, Cambridge, UK; ^8^​ Department of Medicine, University of Cambridge School of Clinical Medicine, Cambridge Biomedical Campus, Cambridge, UK

**Keywords:** antimicrobial resistance, *Corynebacterium diphtheriae*, diphtheria, erythromycin, outbreak

## Abstract

Diphtheria is a potentially fatal respiratory disease caused by toxigenic forms of the Gram-positive bacterium *Corynebacterium diphtheriae *. Despite the availability of treatments (antitoxin and antimicrobials) and effective vaccines, the disease still occurs sporadically in low-income countries and in higher income where use of diphtheria vaccine is inconsistent. Diphtheria was highly endemic in Vietnam in the 1990s; here, we aimed to provide some historical context to the circulation of erythromycin resistant organisms in Vietnam during this period. After recovering 54 *C *. *diphtheriae* isolated from clinical cases of diphtheria in Ho Chi Minh City between 1992 and 1998 we conducted whole genome sequencing and analysis. Our data outlined substantial genetic diversity among the isolates, illustrated by seven distinct Sequence Types (STs), but punctuated by the sustained circulation of ST67 and ST209. With the exception of one isolate, all sequences contained the *tox* gene, which was classically located on a corynebacteriophage. All erythromycin resistant isolates, accounting for 13 % of organisms in this study, harboured a novel 18 kb *erm(X*)-carrying plasmid, which exhibited limited sequence homology to previously described resistance plasmids in *C. diphtheriae *. Our study provides historic context for the circulation of antimicrobial resistant *C. diphtheriae* in Vietnam; these data provide a framework for the current trajectory in global antimicrobial resistance trends.

## Data Summary

1. All supplementary data (Table S1, Table S2, Fig. S1, S2, and S3, available in the online version of this article) have been deposited in FigShare. (https://figshare.com/articles/figure/Supplementary_Figures/14728200)
2. All short reads used in this study were registered in the European Nucleotide Archive (ENA) under study accessions of PRJEB32654 (Accession numbers: ERR3331346- ERR3331399)
3. The plasmid sequence was deposited in GenBank (Accession number: MZ348427)

We confirm that all supporting data, code, and protocols have been provided within the article or through supplementary data files.

**Impact Statement**

Infection caused by *Corynebacterium diphtheriae* has recently become a heavy burden on human health globally. Despite development and global introduction of diphtheria toxoid vaccine, diphtheria, the illness caused by *Corynebacterium diphtheriae* remains endemic in many low-income settings where there are issues with vaccine coverage. The most well-described virulence factor of *C. diphtheriae* is the toxin, which is encoded by the *tox* gene encoded on a beta-corynephage. Diphtheria antitoxin and antimicrobials (such as penicillin and erythromycin) are considered the most effective therapies for the treatment of diphtheria. However, there has been an increase in resistance to these antimicrobials that may limit treatment options. The introduction of the diphtheria vaccine was successful in Vietnam in the 1980s; however, there were several diphtheria outbreaks a decade later, some of which were characterised by a high proportion of erythromycin resistant isolates. In this study, we used whole genome sequencing to investigate the genome structure and diversity of the *C. diphtheriae* population from this period. We identified seven different sequence types (ST) among; the most common was ST67, which has been recently found in outbreaks in the central highlands of Vietnam. The toxigenic and virulence components of these isolates were also characterised. Specifically, we identified a novel plasmid carrying the *erm(X)* gene and conferring erythromycin resistance. Our results highlight the importance of historical collections in providing greater context during contemporary outbreaks and the role of drug resistance.

## Introduction

Diphtheria is a life-threatening upper respiratory disease caused by the Gram-positive organism *Corynebacterium diphtheriae* (* C. diphtheriae *). The organism infects the pharynx, tonsils, and nasal passage, causing a sore throat, low-grade fever, and an inflammatory pseudomembrane on the tonsils 2–3 days post-infection [1, 2]. The most common transmission route of *C. diphtheriae* is via contact with an infected individual or droplets contaminated with the organism [1]. Asymptomatic carriage is common, but infants, the elderly, and the immuno-compromised are at particular risk of severe disease and death (case-fatality rate of >20 %) [2, 3]. Diphtheria toxoid vaccine was developed from the 1920s and introduced into Expanded Programme on Immunization (EPI) internationally in 1977 to reduce *C. diphtheriae* disease and community transmission. However, recent reports highlight that diphtheria remains endemic in several low-income settings. Outbreaks have been reported in India, Laos, Bangladesh and the Yemen and also in high-income settings regions with low immunisation coverage as seen in Norway [4–9]; in 2017 the estimated number of cases globally remained >16 000 [10–12].

Diphtheria toxin is the key virulence factor of *C. diphtheriae* and one of the most well-characterised bacterial toxins. The toxin triggers the characteristic disease features in the respiratory tract and also more severe symptoms including myocarditis, peripheral neuropathy, and acute renal injury [1, 13, 14]. The toxin is encoded by the *tox* gene and is carried on a beta-corynephage [1, 15]. *Tox* is regulated by the metallo-regulatory transcriptional regulator DtxR, which is repressor of tox, and is active when iron is not limited [16]. When secreted, the diphtheria exotoxin induces local tissue destruction and facilitates the formation of the pseudomembrane [1, 15]. Penicillin and erythromycin have long been the primary treatments for diphtheria, but recent reports describe an increase in resistance against these antimicrobials, potentially posing a problem for diphtheria treatment [17–20]. Several studies have descibed resistance mechanisms against antimicrobials used for the treatment of diphtheria [21–23]. Specifically, erythromycin resistance in *C. diphtheriae* is commonly encoded by the plasmid mediated *erm*(X) gene [24]. *Erm*(X) gene encodes an rRNA methyl transferase enzyme catalysing demethylation of adenine in the 23S rRNA gene, which prevents effective binding of the macrolide to the target rRNA [24].

Diphtheria is endemic in Vietnam, although disease incidence declined rapidly after the introduction of the vaccine into the EPI in the late 1980s [25, 26]. The incidence of diphtheria increased in Vietnam in the mid-1990s and there were several outbreaks, with some isolates exhibiting resistant to erythromycin [25, 27, 28]. There are little available data from this period, and no molecular characterisation of bacterial isolates from this period. Here, through whole genome sequencing (WGS) and analysis we aimed to investigate the population structure, diversity, and antimicrobial resistance gene composition in *C. diphtheriae* isolated from Vietnamese patients with symptomatic disease in the 1990s.

## Methods

### Bacterial isolates

We revived 54 *C *. *diphtheriae* isolated from the nose or throat of children with a clinical diagnosis of respiratory diphtheria attending the Hospital for Tropical Diseases in Ho Chi Minh City between 1992 and 1998. Some of them were part of two studies reported in Clinical Infectious Diseases [29, 30]. Briefly, at the time of isolation, throat and nasal swabs from clinical cases were cultured on Hoyles tellurite agar and sheep blood agar and incubated at 37 °C for 24–48 h. Single colonies suspected to be *C. diphtheriae* were identified morphologically using methylene blue and then confirmed by the cysteinase and pyrazinamidase test and by API Coryne (bioMérieux, France) [31]. Toxin production was confirmed by using a standard Elek test. Organisms were stored at −20 °C in protect bottles with the BHI+glycerol 10 % medium until their attempted revival and recovered on sheep blood agar and Brain Heart Infusion broth supplemented with yeast extract (0.4 %) and Tween 80 (0.2 %) (BHI/YE/Tween).

### Antimicrobial susceptibility testing

The Minimum Inhibitory Concentrations (MICs) of the organisms were performed by the agar plate dilution method [32]. Single colonies were sub-cultured into Brain Heart Infusion broth supplemented with yeast extract (0.4 %) and Tween 80 (0.2 %) (BHI/YE/Tween) before incubation at 37 °C for 18 h. A multipoint inoculator (Mast Laboratories Ltd, Merseyside, UK) was used to apply 300 µl inoculum (10^5^ to 10^6^ c.f.u. ml^−1^) of each organism to the surface of Mueller Hinton agar (Unipath Ltd, Basingstoke, UK) containing saponin-lysed sheep blood (5 %) and serial two-fold dilutions of Erythromycin, Tetracycline, Ceftriaxone, Rifampicin, Ampicillin and Chloramphenicol. All antimicrobials were sourced from Sigma Aldrich, except ceftriaxone, which was from Rocephin (Rocephin, Roche, Hong Kong, China). Toxigenic *C. diphtheriae var gravis* NCTC10356, non-toxigenic *C. diphtheriae var gravis* NCTC11397, and *Staphylococcus aureus* ATCC25923 were included as controls. Plates were incubated at 37 °C for 18 h. Antimicrobial susceptibility was determined following guideline for infrequently isolated or fastidious bacteria [33].

### Genome sequencing

Genomic DNA from all isolates was extracted by using the Wizard Genomic DNA Extraction Kit (Promega, USA) [34] and quality assessed using a Qubit dsDNA system (Invitrogen) [35]. The genomic DNA was diluted and 1 ng from each isolate was used for library preparation in accordance with the guidelines of the manufacturer (New England Biolab and NexteraXT) before being subjected to WGS on an Illumina Miseq System [36]. FastQC was used for assessing the quality of reads and passed reads were progressed for mapping and assemblies [37]. The FASTQ generated were submitted to the ENA (https://www.ebi.ac.uk/ena) under the project number of PRJEB32654, and accession numbers from ERR3331346 to ERR3331399 (Table S1).

### Genome analysis

All reads passing QC checking were mapped to the NCTC13129 reference genome (Accession no. BX248353.1) using Bowtie2 v2.2.9 and SAMTools v1.3.1. All single nucleotide polymorphisms (SNPs) with the Phred quality score of ≥30 of each isolate were determined and called [38, 39]. A total of 66 761 conserved SNPs were identified after removing those located in the recombinogenic regions and those arising in <5 % of all isolates [40, 41]. The pseudogenome alignment was generated and then subjected to statistical scanning for potential recombinations using Gubbins. These regions were excluded from further analysis [42]. Variable sites that had minimum read depth ≤5 and a genome coverage ≤50 % were also excluded.

A phylogeny of 54 *C *. *diphtheriae* genome sequences was reconstructed from core SNPs determined from mapping using Randomized Accelerated Maximum Likelihood (RAxML) [43]. RAxML was run five times for SNP allele table by using the generalized time-reversible model with Γ distribution (GTR**+**Γ) to model site-specific rate variation with one hundred bootstrap pseudo-replicate analyses to assess the maximum likelihood (ML) tree supports.

Known alleles associated with antimicrobial resistance (AMR) and virulence genes were directly detected from read sets mapping approach based on SRST2 [26]. The ARG-Annot database was used for the detection of AMR genes. MLST and virulence genes were determined using the *C. diphtheriae* BIGSdb database at Institute Pasteur (http://bigsdb.web.pasteur.fr) and Virulence Factors Database (VFDB) for Corynebacterium. Additionally, fastq reads were further *de novo* assembled using SPAdes v3.14.1 to generate contigs. Contaminated sequences were removed according to the size of contigs compared that of the reference genome. Genomes with evidence of contamination were excluded from the study, according to the following criteria: total assembly length >2.5–3 Mb, with evidence of >1 % read contamination as determined by MetaPhlAn and applied for other MDR bacteria, or <50 % reads mapping to the NCTC13129 reference chromosome (accession number: BX248353.1); or a ratio of heterozygous/ homozygous single nucleotide polymorphism (SNP) calls compared to the reference chromosome exceeding 20 % [44] . The presence of AMR carrying plasmid was resolved and visualized using assembled contigs in Bandage v.0.8.1 [45].

### Plasmids

Isolates carrying the *erm*(X) gene were cultured on sheep blood agar supplemented with Erythromycin (2 mg l^−1^) and plasmid DNA was extracted using the Plasmid Miniprep Kit (QIAprep Kit). Plasmid DNAs were subjected to library preparation prior to sequencing on an Illumina Miseq system. All raw reads were *de novo* assembled and visualized in Bandage to determine and resolve the *erm*(X) carrying plasmid. The circularised plasmid was compared with the reference plasmid pNG2 (Acession no. AF492560.1) using Blast Ring Image Generator (BRIG) v0.95 [46]. The plasmid sequences were deposited in Genbank (Accession number: MZ348427).

## Results

### Genomic diversity of *C. diphtheriae*

**Fig. 1.**
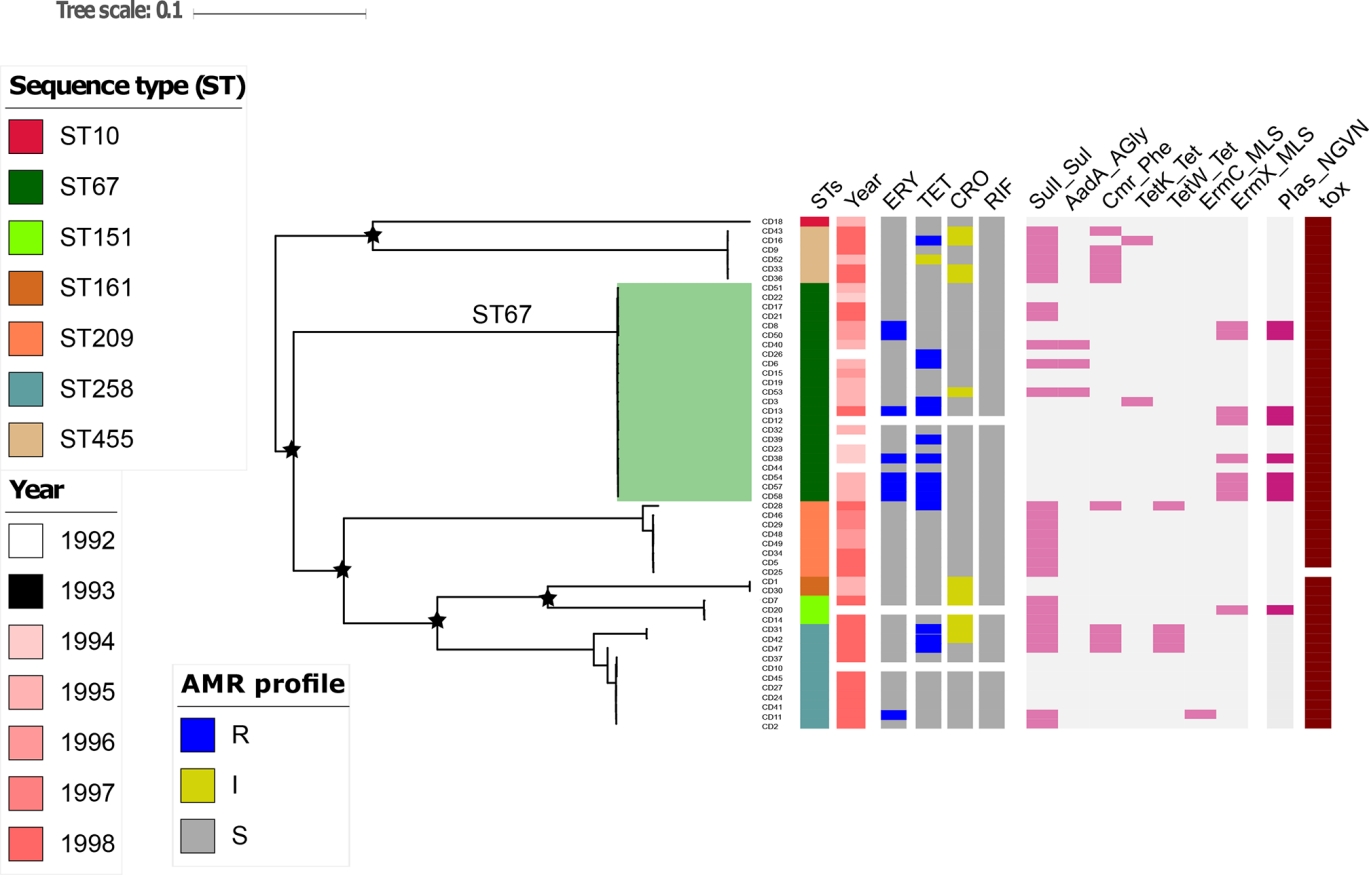
Phylogeny, antimicrobial resistance phenotype and genotype of the *C. diphtheriae* isolates. Maximum likelihood phylogeny of 54 *C *. *diphtheriae* was reconstructed from 66 761 core SNPs. The corresponding columns were metadata: Sequence Type (STs), the year of isolation (Year), antimicrobial susceptible profile (blue: resistant; yellow: intermediate; grey: susceptible) using agar plate dilution method for Erythromycin (ERY), Tetracycline (TET), Ceftriaxone (CRO), Rifampicin (RIF). The heat map was the antimicrobial resistant genes (pink: gene presence; green: gene absence) and the last columns was a determined plasmid carrying *erm*(X) gene encoding for erythromycin resistance (pink: plasmid presence; green: plasmid absence). The stars show bootstrap support values 100 % on internal nodes. The shading colour was noted for the most common sequence type of ST67.

Of the 54 recovered *C. diphtheriae* isolated from 1992 to 1998 from patients with diphtheria, the overwhelming majority (53/54; 98.15 %) were confirmed to be toxigenic (Table S1). Mapping reads from the WGS of 54 isolates to the *C. diphtheriae mitis* NCTC13129 reference genome determined an average coverage of 90.7 %, an average mapping of 86.19 %, and an average read depth coverage of 59×. A phylogenetic tree was reconstructed from 66 761 core SNPs and identified seven independent lineages. Multilocus sequence typing (MLST) analysis delineated seven independent STs; of which ST67 was the most common (23/54; 42.6 %), followed by ST258 (11/54; 20.4 %), ST209 (8/54; 14.8 %), ST455 (6/54, 11.1 %), ST151 (3/54, 5.5 %), ST161 (2/54, 3.7 %) and ST10 (1/54, 1.8 %) (Fig. 1).

We aligned supplementary metadata with the phylogeny, which included year of sampling and antimicrobial susceptibility against to erythromycin, tetracycline, ceftriaxone, and rifampicin. All isolates were susceptible to rifampicin (MIC ≤0.016 mg l^−1^). Almost all isolates were susceptible to ceftriaxone (MIC ≤1.0 mg l^−1^), with 20 % (11/54) exhibiting intermediate resistance (MIC=2 mg l^−1^). Notably, 13 % (7/54) of isolates were resistant to erythromycin, the traditional second line antimicrobial for diphtheria treatment. The proportion of isolates that were resistant to tetracycline, an alternative second line antimicrobial, was 18.5 % (10/54). The sequences were screened for genes encoding resistance to antimicrobials; 16.7 % (9/54) of organisms carried *erm*(X), conferring resistance to erythromycin (Fig. 1). In addition, half of the sequenced isolates possessed the *sul*1 gene, encoding sulphonamide resistance, and 16.7 % (9/54) of sequences carried the *cmr* gene, encoding resistance to chloramphenicol, correlating with an MIC ≥2 mg l^−1^ against chloramphenicol. The MIC values against ampicillin ranged from 0.06mg l^−1^ to 1 mg l^−1^.

### Virulence factors of *C. diphtheriae*

Three distinct pili structures have been described in *C. diphtheriae* [47, 48]. These structures are encoded by three different gene clusters and facilitate the attachment of the organism to host cells. Analysis of the three pilus encoding regions in our *C. diphtheriae* genomes collection found that all organisms lacked the SpaH-type pilus (Fig. S1); however, most isolates (96.3 %; 52/54) possessed the SpaA-type pilus. Additionally, the SpaD-type pilus was conserved in all the ST455, ST10, and ST161 isolates only. The SapD surface-anchored pilus proteins were present in all isolates, whereas only six isolates of ST455 contained SapA.

**Fig. 2.**
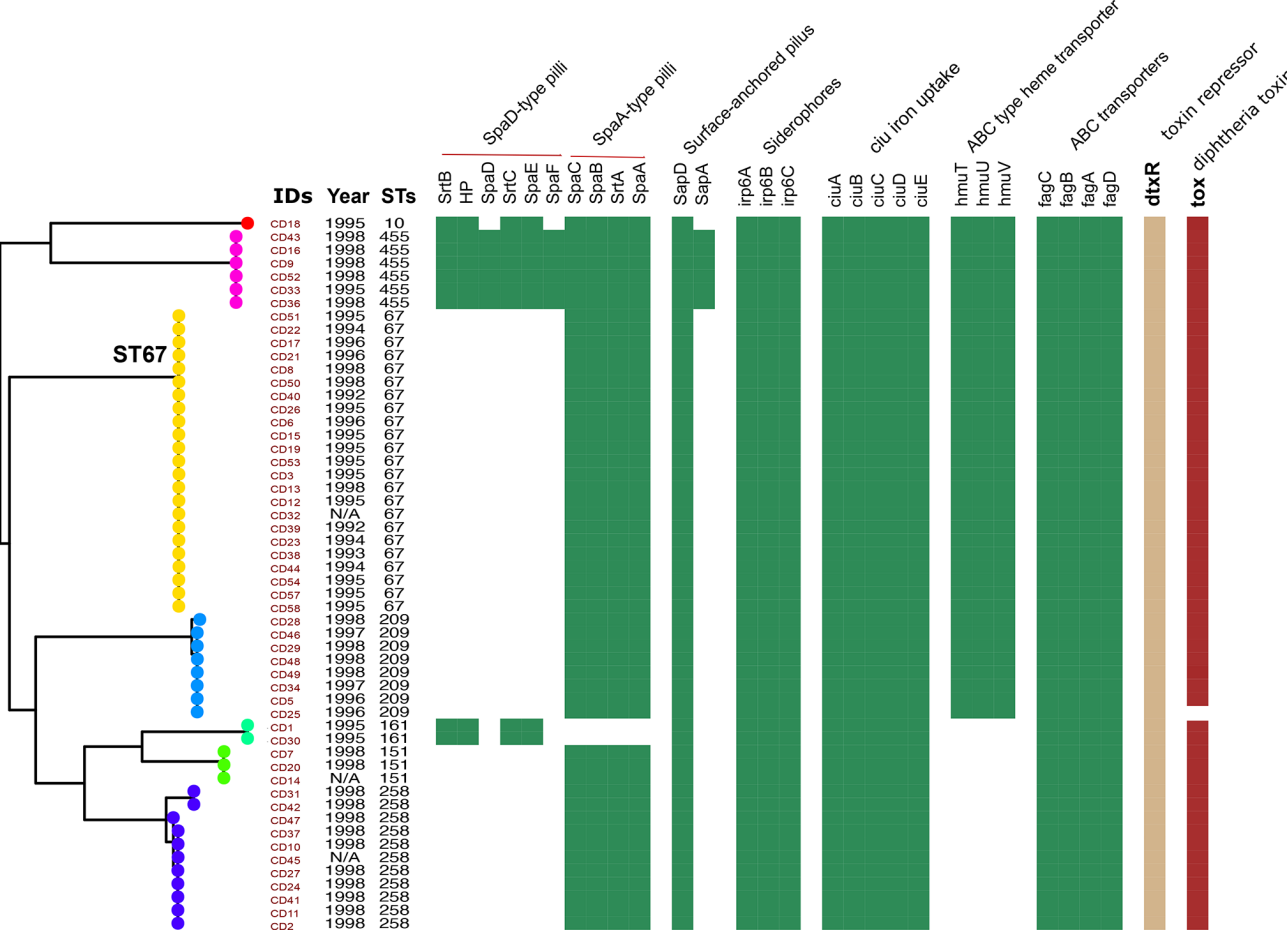
Virulence factors in the 54 *C *. *diphtheriae* isolates. SpaD and SpaA-type pili gene clusters encoding for pili proteins that were employed to target to human pharyngeal cells. STs: sequence types of isolates. Colours: gene presence; white: gene absence.

Other gene clusters related to iron uptake was present in all isolates, with the exception of ABC-type haem transporters which were lacking in the ST161, ST151 and ST258 organisms (Fig. 2). In concordance with the phenotypic data, we found almost all (98.1 %; 53/54) organisms possessed *tox* (associated with the corynebacteriophage). Notably, the non-toxigenic isolate missing the *tox* gene was also lacking the entire prophage (Fig. S2). Despite the prophage being comparatively well conserved in the ST67 and ST258 isolates, the genes encoding the tail proteins and prophage anti-repressor were absent. Other organisms in the collection were found to be lacking some regions encoding exported proteins and various transcriptional regulators (Table S2).

### A novel plasmid carrying *erm(X*) gene

**Fig. 3.**
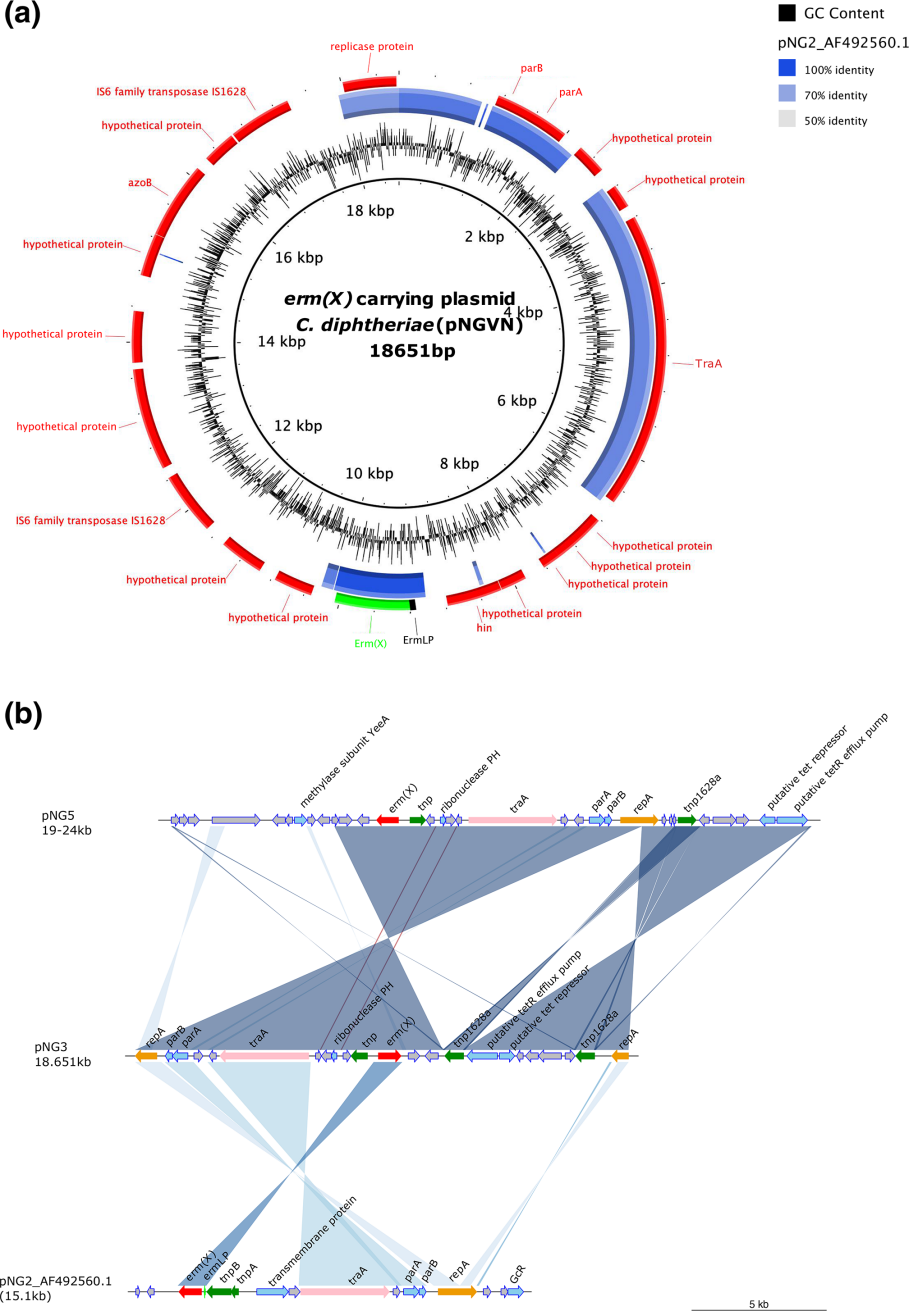
The genetic structure of *erm(X*)-carrying plasmids in *C. diphtheriae *. **a.** Pairwise comparison of *erm*(X)-carrying plasmids: described plasmid (pNG2, AF_492560.1, 15 kb) and our novel plasmid (pNGVN, 18.651 kb) detected in six out of nine *C. diphtheriae* strains and a larger plasmid (pNG5, 19–24 kb) possessed by three other isolates. **b.** The genetic structure of the *erm(X*)-carrying plasmid pNGVN. blastn comparison between pNGVN (central ring) identified from an erythromycin resistant ST67 outbreak isolate and pNG2 plasmid from GenBank (Accession number: AF_492560.1). The outermost ring indicates the gene annotations of the pNGVN plasmid. *erm(X*) gene is highlighted in green colour.

To better characterise the plasmid carrying the *erm*(X) gene, we performed a plasmid DNA extraction and sequenced the raw plasmid DNA. Reads were *de novo* assembled and generated three large contigs with a coverage of 248×, 235×, and 480×, respectively (Fig. S3). Comparing the contigs from the plasmid with 480× coverage with sequences available on GenBank, we identified that these contigs encoded an 842 bp mobile element. Using Bandage to create an interactive visualization of the assembly graph from the *de novo* assembly, we resolved these contigs and assembled a novel circular plasmid of 18 651 kb, which we named pNGVN (Fig. 3b). A pairwise comparison between pNGVN and previously described plasmid pNG2 (AF_492560.1) revealed that the pNGVN had 65 % sequence coverage and an average of identity of 88.78 % (85.44–98.23 %) to pNG2 (Fig. 3a). We described a total of 23 putative coding sequences in pNGVN; of which 10/23 coding sequences were functionally annotated. Plasmid pNGVN contained several functional genes that were comparable to those of pNG2 including *rep*A, *tra*A, *par*A, and *par*B. These genes are a replicase protein, a conjugal transfer protein, and type Ib plasmid partitioning proteins, respectively. Additionally, the *erm*(X) gene on pNGVN was 98 % identical to that on pNG2 and located next to transposable elements that were previously identified in *C. striatum* and *C. resistans*. In the pNG2 plasmid sequence, the *erm*(X) gene had an an *erm*(LP) located upstream. This gene was annotated as the 23S rRNA adenine N-6-methyltransferase leader peptide. We also identified an identical sequence to the *erm*(LP) gene in pNGVN; however, the output from the Prokka tools for our sequence was unable to annotate this gene. Six out of nine organisms carrying *erm*(X) possessed pNGVN like plasmids. The additional three isolates possessed a larger plasmid (pNG5, 19–24 kb) with a pNGVN backbone with an additional cassette gene (~6 kb) carrying the methylase subunit, *Yee*A and a further nine hypothetical proteins (Fig. 3a).

## Discussion

Here, we sequenced whole genome of 54 *C *. *diphtheriae* isolates collected from patients admitted to a major tertiary hospital in the south of Vietnam in the 1990s. Our results outlined substantial genetic diversity among the Vietnamese *C. diphtheriae* population, with seven different STs identified. The most common STs (ST67) and the ST209 have also been identified in diphtheria outbreaks in the western and central highlands of Vietnam recently, implying the sustained circulation of these STs across the country [28]. ST67 was the most common genotype associated with diphtheria in an observational studies from San Lazaro Hospital in the Philippines and in Europe [49, 50], whereas ST258 and ST209 were both reported in Thailand in 2012 [51]. Estimating within-group SNP variation for different STs in our study showed SNP numbers varied substantially within these STs; the average of SNP difference in ST67 was 65 (range: 51-83 SNPs), 39 in ST151 (range: 32-49 SNPs), 32 in ST258 (range: 29-36 SNPs) and 20 in ST455 (range: 17-26 SNPs). There are different thresholds for genetic associations suggesting whether organisms originated from outbreaks. The threshold for a recent diphtheria outbreak in the US was 11.6 SNPs (range: 0-24 SNPs) and 2–12 SNPs has been suggested for other MDR bacteria [52–55]. The diverse nature of the organisms within this study suggest that these organisms were indicative of endemic circulation rather than an outbreak.

Almost all *C. diphtheriae* isolates in this were susceptible to rifampicin and ceftriaxone (some organisms higher MICs at 2 mg l^−1^), whilst 13 and 18.5 % of isolates were resistant to erythromycin and tetracycline, respectively. The organisms described generally exhibited lower MIC (0.06 mg l^−1^ ≤MIC ≤1 mg l^−1^) against chloramphenicol than those described by Maple *et al*. (all at MIC at 1 mg l^−1^) [56]. Notably, despite these organisms being isolated in the 1990s, the prevalence of erythromycin resistance was higher than the reports from Indonesia and Brazil (5.3 %, 4.2 %, respectively). Alternatively, the observed proportion of tetracycline resistance was higher than in the aforementioned study in Brazil (12.8 %), but substantially lower than that described in Indonesia (84.2 %) [17, 18]. Screening for AMR genes identified a variety of genes conferring AMR phenotypes. All isolates were susceptible to penicillin; therefore, we found no pbp2m gene or presence of pLRPD-like plasmid in this collection. Although some organisms had higher MICs against ceftriaxone (MIC=2 mg l^−1^), we did not detect any genes/chromosomal mutations associated with this phenotype. A principal observation was the identification of the *erm*(X) gene, encoding erythromycin resistance located on a novel 18 651 kb plasmid (primarily found in the CD54 strain). The plasmid exhibited 65 % DNA sequence identity to plasmid pNG2 (AF_492560.1, 15 kb), which was described by Tauch and colleagues in 2003. In total we identified 23 coding sequences from the novel plasmid, 47.8 % of these had been functionally annotated, necessitating some further work to determine the function of the identified sequences.

We additionally found that almost all isolates (98 %) were toxigenic, which was associated with the *tox* gene located on the classical beta corynephage. However, we did identify some signs of genetic degradation around the *tox* gene, with genes encoding bacteriophage tail proteins, prophage anti-repressor, exported and transcriptional proteins were missing. To our knowledge this is the first time such degradation has been observed around the *tox* gene, but genetic variation with the corynephage sequence is relatively common [57–59]. Additionally, most isolates possessed the SpaA pilus. The presence of the SpaA type pili had been determined to be essential for corynebacterial adherence to the host cells and known as pilus mediated adherence. The SpaD-type pili was identified in only nine isolates, while SpaH was not detected [60]. This has also been observed in other *C. diphtheriae* and is therefore not unique to these isolates from Vietnam. These data suggest some redundancy in the pili required to attach to host cells and induce infection of the pathogen. However, we did not perform phenotypic characterisation and the loss (or rearrangement) of some genetic material may be an artefact of long term storage after the original isolation, which is a potential limitation.

Diphtheria has been largely controlled by the sustained use of the vaccine since the 1980s; however, in locations where vaccine uptake is less complete the organism still has the potential to trigger outbreaks. This scenario is more common in low-middle income countries, such has Vietnam, where the cases of diphtheria arise sporadically for sustained periods and ongoing surveillance is required. Here, using a historic collection of organisms and data we have investigated a collection of *C. diphtheriae* isolated in Vietnam in the 1990s, when erythromycin resistance was an emerging problem. Our data provide some additional context to *C. diphtheriae* in Vietnam and we can show that identical STs (67 and 209) have remained in circulation for >30 years and are capable of seeding new outbreaks [28]. Our results emphasize that historic collections of organisms improve the framework for ongoing surveillance for diseases that have not yet been eradicated and provide better context for the current trajectory in global antimicrobial resistance trends.

## Supplementary Data

Supplementary material 1

Supplementary material 2

Supplementary material 3

Supplementary material 4

Supplementary material 5
