# Supplementary material for: Endemic erythromycin resistant Corynebacterium diphtheriae in Vietnam in the 1990s
Source: Microb Genom. 2022 Oct 19;8(10):mgen000861. doi: 10.1099/mgen.0.000861 (PMC9676054; doi:10.1099/mgen.0.000861)
Supplement: Supplementary material 1 [file mgen-8-861-s001.pdf]

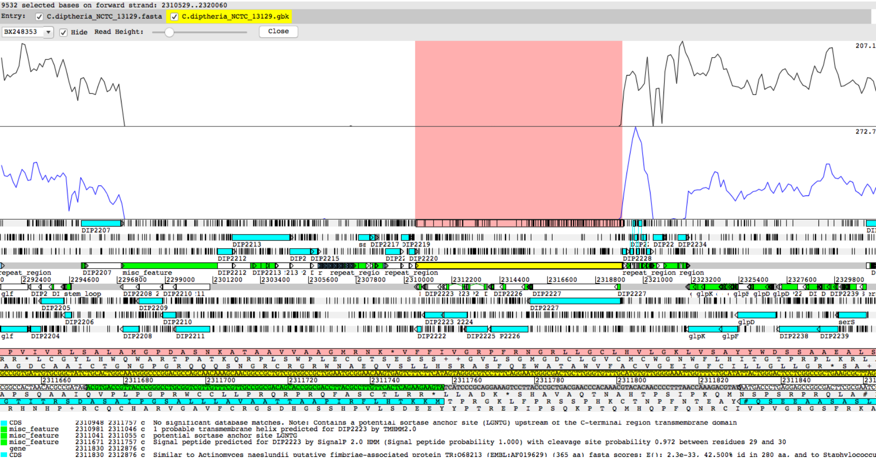

9532 selected bases on forward strand: 2310529..2320060
Entry: C.diptheria_NCTC_13129.fasta
C.diptheria_NCTC_13129.gbk
BX248353
Hide
Read Height:
Close
207.1
272.7
CDS 2310948 2311757 c No significant database matches. Note: Contains a potential sortase anchor site (LGNTG) upstream of the C-terminal region transmembrane domain
misc_feature 2310981 2311046 c 1 probable transmembrane helix predicted for DIP2223 by TMHMM2.0
misc_feature 2311041 2311055 c potential sortase anchor site LGNTG
misc_feature 2311671 2311757 c Signal peptide predicted for DIP2223 by SignalP 2.0 HMM (Signal peptide probability 1.000) with cleavage site probability 0.972 between residues 29 and 30
gene 2311830 2312876 c
CDS 2311830 2312876 c Similar to Actinomyces naeslundii putative fimbriae-associated protein TR:O68213 (EMBL:AF019629) (365 aa) fasta scores: E(): 2.3e-33, 42.500% id in 280 aa, and to Staphylococcu
