# Supplementary figures and images for: Endemic erythromycin resistant Corynebacterium diphtheriae in Vietnam in the 1990s
Source: Microb Genom. 2022 Oct 19;8(10):mgen000861. doi: 10.1099/mgen.0.000861 (PMC9676054; doi:10.1099/mgen.0.000861)

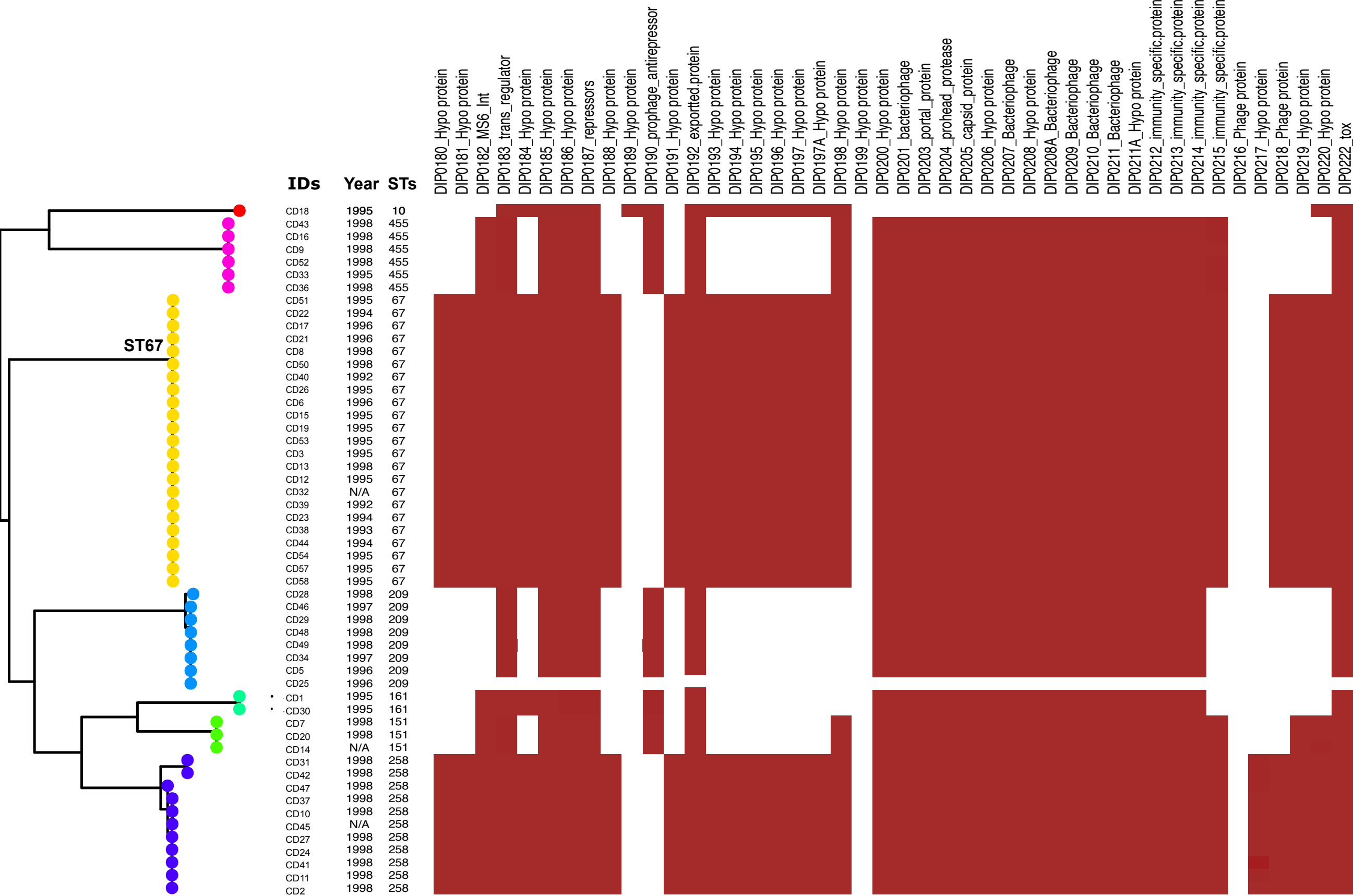

Supplement: Supplementary material 2 [file mgen-8-861-s002.pdf]

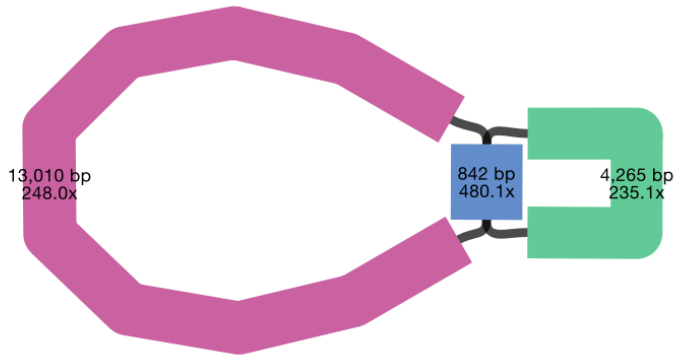

Supplement: Supplementary material 3 [file mgen-8-861-s003.pdf]
